# The game theory of *Candida albicans* colonization dynamics reveals host status-responsive gene expression

**DOI:** 10.1186/s12918-016-0268-1

**Published:** 2016-03-01

**Authors:** Katarzyna M. Tyc, Sanna E. Herwald, Jennifer A. Hogan, Jessica V. Pierce, Edda Klipp, Carol A. Kumamoto

**Affiliations:** Theoretische Biophysik, Humboldt-Universität zu Berlin, Invalidenstraße 42, D-10115 Berlin, Germany; Graduate Program in Molecular Microbiology, Sackler School of Graduate Biomedical Sciences and Department of Molecular Biology and Microbiology, Tufts University, Boston, MA 02111 USA; Department of Molecular Biology and Microbiology, Tufts University, 136 Harrison Ave., Boston, MA 02111 USA; Present address: Department of Biochemistry, University of Utah, Salt Lake City, UT 84112 USA; Genetics and Biochemistry Branch, National Institute of Diabetes and Digestive and Kidney Diseases, National Institutes of Health, Bethesda, MD 20892 USA

**Keywords:** *Candida albicans*, Colonization, Fungal pathogen, Microbiota, Efg1p, Evolutionary game theory, Mathematical modeling

## Abstract

**Background:**

The fungal pathogen *Candida albicans* colonizes the gastrointestinal (GI) tract of mammalian hosts as a benign commensal. However, in an immunocompromised host, the fungus is capable of causing life-threatening infection. We previously showed that the major transcription factor Efg1p is differentially expressed in GI-colonizing *C. albicans* cells dependent on the host immune status. To understand the mechanisms that underlie this host-dependent differential gene expression, we utilized mathematical modeling to dissect host-pathogen interactions. Specifically, we used principles of evolutionary game theory to study the mechanism that governs dynamics of *EFG1* expression during *C. albicans* colonization.

**Results:**

Mathematical modeling predicted that down-regulation of *EFG1* expression within individual fungal cells occurred at different average rates in different hosts. Rather than using relatively transient signaling pathways to adapt to a new environment, we demonstrate that *C. albicans* overcomes the host defense strategy by modulating the activity of diverse fungal histone modifying enzymes that control *EFG1* expression.

**Conclusion:**

Based on our modeling and experimental results we conclude that *C. albicans* cells sense the local environment of the GI tract and respond to differences by altering *EFG1* expression to establish optimal survival strategies. We show that the overall process is governed via modulation of epigenetic regulators of chromatin structure.

**Electronic supplementary material:**

The online version of this article (doi:10.1186/s12918-016-0268-1) contains supplementary material, which is available to authorized users.

## Background

By colonizing the human gastrointestinal tract as a component of the normal microbiota [1], the opportunistic fungal pathogen *Candida albicans* establishes a reservoir from which it can disseminate to cause systemic candidiasis. In an immunocompromised patient, the fungus is capable of escaping from the gastrointestinal (GI) tract, reaching the bloodstream and infecting deep organs, producing a life-threatening disseminated infection [2–8]. In contrast, in an immunocompetent host, the organism remains as a benign commensal within the gut. Colonization of the GI tract is controlled by interaction between the host’s immune system [8–19], resident intestinal microbiota [5, 6, 20–23], and the fungus [24–29].

We previously identified *C. albicans* transcription factor Efg1p as a regulator of GI colonization [26, 30]. Furthermore, in wild type (WT) *C. albicans* cells recovered from organs of infected mice, mRNA levels of *EFG1* in cells colonizing immunodeficient mice were lower than levels in cells colonizing immunocompetent mice [26]. Since Efg1p is an important regulator of *C. albicans* physiology [31–35] and gene expression [34, 36–40], the difference in its expression may represent an important first step in the progression toward disease in the compromised host.

In this communication, we present a mathematical model that describes the dynamics of colonization by *C. albicans* cells expressing either high or low levels of *EFG1*. The goal of the work was to demonstrate the existence of host-responsive mechanisms that regulate the expression of *EFG1* during colonization. We employed principles of evolutionary game theory to model this host-pathogen interplay. Game theory is a mathematical way to study decision-making and has previously been applied to study biological systems, for instance, when studying *C. albicans* survival strategies upon macrophage attack [41]. Evolutionary game theory is applied to biological systems to predict outcomes based on the frequency and efficacy of different strategies present within a population. Game theory breaks a situation down into a set of interacting entities, termed players, a set of strategies available to those players, and a payoff to each player based on each combination of strategies. In evolutionary game theory, the payoffs represent relative differences in fitness, which influence reproduction and survival. Using these principles we constructed a game theoretical model to describe the interaction between *C. albicans* and its host.

Results from our modeling approach strongly support a model where at least two mechanisms contribute to shaping the *C. albicans* population. First, selective forces from the GI tract environment favor cells with higher Efg1p activity; this selective force is reduced in an immunodeficient host. This mechanism does not require a response of *C. albicans* to the host environment. Second, individual cells change from high Efg1p activity to low Efg1p activity in response to the GI tract environment. In this case, a difference in the environment is sensed, resulting in a change in gene expression. We demonstrate that during colonization of the host, *C. albicans* regulates the expression of several activities that control chromatin structure. Our data, in combination with mathematical modeling, strongly suggest that *C. albicans* responds to immune activities of the host by establishing optimal survival strategies via altering the machinery that controls gene expression.

## Results

### Modeling based on evolutionary game theory

**Fig. 1:**
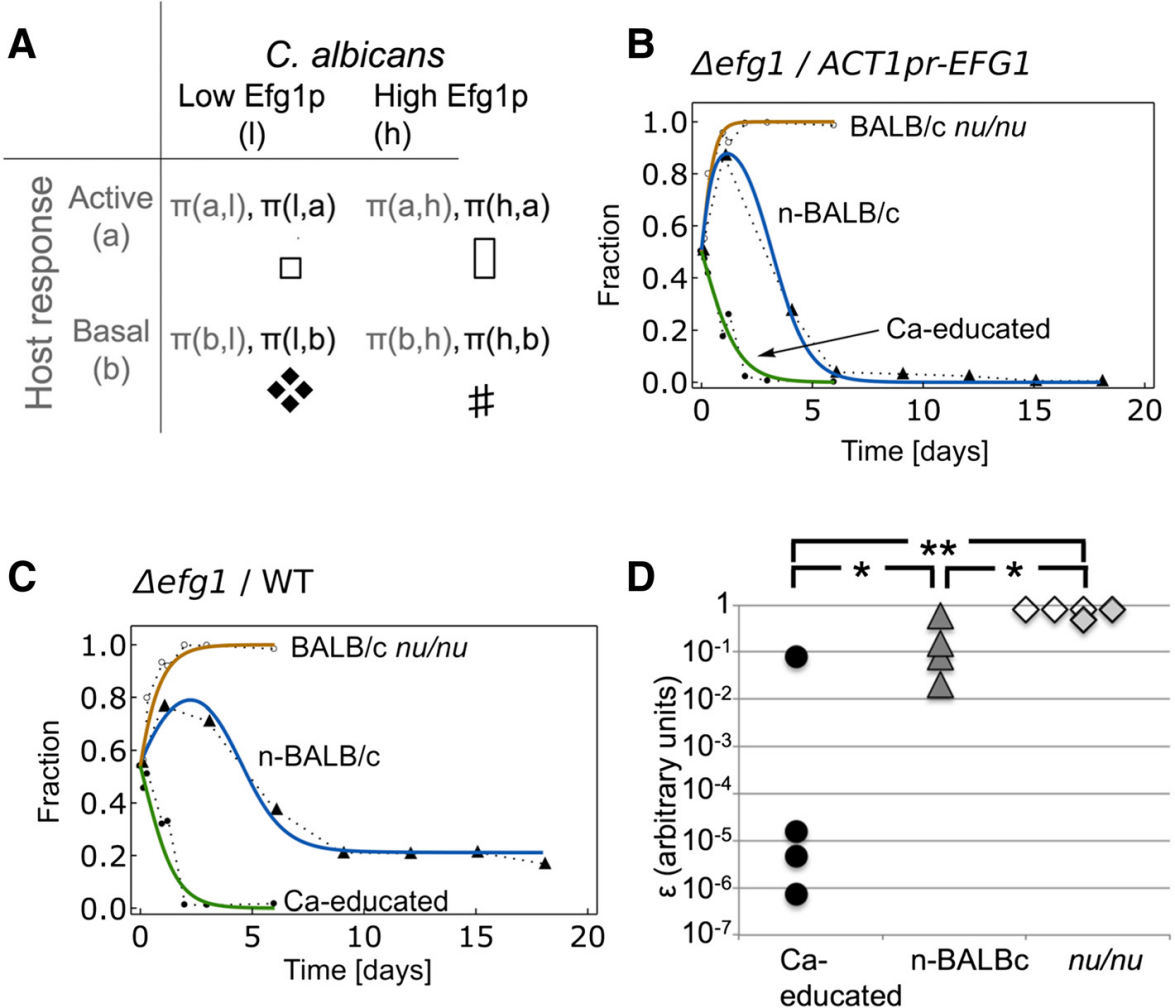
Model simulations of *C. albicans* population dynamics follow experimental results. Panel **a**: Payoffs for *C. albicans* (black letters or pictures) and host cells (grey letters) as a result of their interactions are illustrated in the table. For each combination of fungal and host cell strategies, there is a payoff to the fungal cell (shown as a picture) and a payoff to the host cell (not depicted). Panels **b** and **c**: The fraction of low-*EFG1* cells is plotted as a function of time of fecal pellet collection. Averaged experimental data are shown as symbols connected by dotted lines. Open circles, BALB/c *nu/nu* mice; closed triangles, n-BALB/c mice; closed circles, Ca-educated BALB/c mice. Simulations generated with the model are shown in bolded lines. Orange, BALB/c *nu/nu* mice; blue, n-BALB/c mice; green, Ca-educated BALB/c mice. Panel **b**: Competition between Δ*efg1* null mutant and *ACT1pr*-*EFG1* strains of *C. albicans*. Values for parameters (in arbitrary units) were as follows: for the hosts *π(a,l)* = 1.75, *π(b,l)* = 0.00071, *π(a,h)* = 9.25, *π(b,h)* = 0.0063, and for fungi *π(l,a)* = 0.12, *π(l,b)* = 3.33, *π(h,a)* = 1.34, *π(h,b)* = 0.0067. Panel **c**: Competition between Δ*efg1* null mutant and WT *C. albicans* strains. Values for parameters were: for the hosts *π(a,l)* = 1.75, *π(b,l)* = 0.00071, *π(a,h)* = 0.086, *π(b,h)* = 0.12, and for fungi *π(l,a)* = 0.12, *π(l,b)* = 3.33, *π(h,a)* = 1.47, *π(h,b)* = 2.91; *ε*_Ca-educated_ = 2.57 x 10^−5^, *ε*_n-BALB/c_ = 2.86 x 10^−1^, *ε*_*nu/nu*_ = 9.65 x 10^−1^. Panel **d**: Using the data from individual mice, values for *ε* (in arbitrary units) were extracted. Each symbol indicates the average value of *ε* for colonization of an individual mouse. Ca-educated BALB/c mice (black circles); n-BALB/c mice (grey triangles); BALB/c *nu/nu* mice (open diamonds); Ca-educated BALB/c *nu/nu* mice (grey diamonds). *, *p <* 0.05; **, *p <* 0.01

We previously reported that *EFG1*, encoding a basic helix-loop-helix (bHLH) transcription factor, is differentially regulated in hosts with varying immune activity [26]. To study the mechanisms that gave rise to differences in *EFG1* expression during colonization, we undertook a quantitative analysis of colonization data using mathematical modeling based on the principles of evolutionary game theory. In this model, the players are *C. albicans* and the host and each has two available strategies (Additional file 1: Figure S1A). The strategies available to *C. albicans* cells are high Efg1p activity (denoted *h*) and low Efg1p activity (denoted *l*). The strategies available to the host are basal (denoted *b*) or activated (denoted *a*). As a result, there are 4 possible combinations of individual fungal cell and host cell strategies (Fig. 1a). For each fungal cell and host cell undergoing an interaction, there is a payoff value, π, which depends on the combination of fungal and host strategies. The values for the 4 payoffs, π, to *C. albicans* cells (Fig. 1a) depend on the strategy chosen by the fungal cell and the host cells. Similarly, there are 4 payoffs, π, to the host (Fig. 1a) that depend on the strategy used by the host cell and the *C. albicans* cell.

Within an individual host, changes in fungal and host cell populations during colonization (Additional file 1: Figure S1) were modeled using a set of ordinary differential equations that incorporated the 4 different payoffs to *C. albicans* and the 4 different payoffs to the host. The following equation was used to describe the *C. albicans* population during colonization:

1 $$ {\overset{.}{x}}_l={x}_l\left(1-{x}_l\right)\left[\overline{\pi}\left(l,y\right)-\overline{\pi}\left(h,y\right)\right]+\varepsilon \left(1-{x}_l\right) $$

where *x*_*l*_ denotes the proportion of low Efg1p activity cells in the population, *y* denotes the proportion of host cells that are activated or basal (*y*_a_ or *y*_b_), $\overline{\pi}\left(i,y\right)$ denotes the average payoffs to *C. albicans* cell type *i* (either *l* or *h*) depending on host strategy *j* (either *b* or *a*) corrected for the fraction of the host population using that strategy (*y*_*j*_), and *ε* denotes the rate at which *C. albicans* cells change from high Efg1p activity to low Efg1p activity. The first terms on the right side of Eq. 1 relate to the proportion of cells with low and high Efg1p activity (with *x*_*h*_ = 1-*x*_*l*_), the expression in square brackets relates to differences in average payoffs and the last term takes into account changes of individual *C. albicans* cells from high to low Efg1p activity. The derivation of this equation is based on [42] and described in Additional file 2.

Similarly, the following equation was used to model the responses of the host to *C. albicans* during colonization:

2 $$ {\overset{.}{y}}_a={y}_a\left(1-{y}_a\right)\left[\overline{\pi}\left(a,x\right)-\overline{\pi}\left(b,x\right)\right] $$

In Eq. 2, *y*_*a*_ denotes the proportion of host cells using the activated immune response strategy, *x* indicates the proportion of fungal cells that are in the low or high Efg1p activity state (*x*_*l*_ or *x*_*h*_), and $\overline{\pi}\left(j,x\right)$ are the average payoffs to the host cells depending on the strategy of the *C. albicans* cells corrected for the fraction of the fungal population using that strategy. The derivation of this equation is analogous to Eq. 1. The final output of this model will be a description of the proportion and type of cells in the fungal and host populations over time during colonization.

### *In vivo* colonization dynamics data used for the modeling

Our evolutionary game theory model was partially supported with published *in vivo* data [26]. In addition, we generated a new set of data that reports on *C. albicans* colonization in mice with differing levels of immune response. These experiments were specifically designed for the needs of the model. We measured changes in fungal populations that occurred during colonization of either immunodeficient mice or previously challenged, “Ca-educated” mice. We considered competition between *C. albicans* cells with no *EFG1* expression versus *C. albicans* cells with *EFG1* expression in mice with three levels of immunity: 1) naive immunocompetent mice, with no experience of *C. albicans* colonization, referred to as n-BALB/c mice; 2) “Ca-educated” BALB/c mice, which exhibit a restrictive GI tract environment because they were previously challenged with *C. albicans;* 3) BALB/c *nu/nu* mice, which are immunodeficient due to their lack of T cells.

Ca-educated mice were produced by oral inoculation of BALB/c mice with *C. albicans,* which results in protective immune responses against orally or intravenously inoculated *C. albicans* [11, 43–50]. Ca-educated BALB/c mice were used because their GI tract environment was “restrictive” due to the immune response of the host, the effects of microbiota or both. A restrictive GI tract environment has previously been shown to favor *EFG1-*expressing cells over cells lacking Efg1p [26] which was a foundation for our modeling studies. BALB/c *nu/nu* mice are unable to suppress fungal colonization, carry a different microbiota [21] and have a “permissive” GI tract environment. Under the conditions of these experiments, *nu/nu* mice did not develop deep-seated or mucosal fungal infection, but were preferentially colonized by low-*EFG1-*expressing *C. albicans* populations [26].

In the studies described here, mice were inoculated by oral gavage with equal numbers of *Δefg1* cells and cells either overexpressing *EFG1* under the *ACT1* promoter (denoted by *ACT1pr-EFG1*) or WT cells expressing *EFG1* from the native promoter. Following inoculation with either of the mixes, relative colonization was monitored in fecal pellets (see Materials and Methods), which was previously shown to correlate well with colonization measured in the lower GI tract [28].

**Fig. 2:**
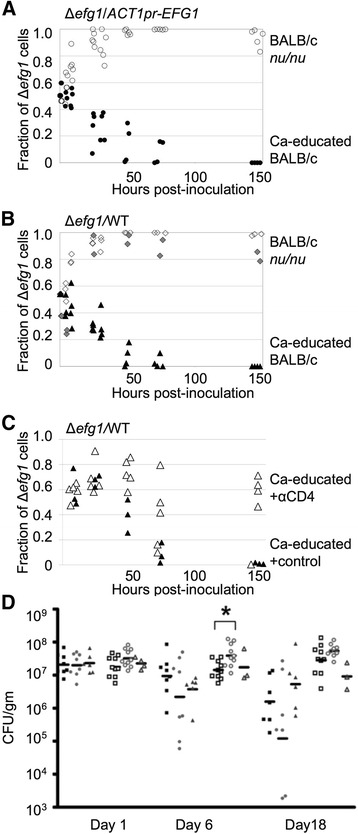
Competition between cells with or without *EFG1*. Colonization dynamics of mixed populations of *C. albicans* were measured in Ca-educated mice (black circles or triangles) or BALB/c *nu/nu* mice (open or grey circles or diamonds). Panels **a**, **b**, and **c** show the fraction of Δ*efg1* null mutant cells in either of the mixed populations in fecal pellets as a function of time. Each symbol indicates a sample from an individual mouse. Panel **a**: Competition between Δ*efg1* and *ACT1pr-EFG1* cells. Closed circles, Ca-educated BALB/c mice. Open circles, BALB/c *nu/nu* mice. Panel **b**: Competition between Δ*efg1* and WT cells. Closed triangles, Ca-educated BALB/c mice. Open diamonds, BALB/c *nu/nu* mice. Grey diamonds, Ca-educated BALB/c *nu/nu* mice. Panel **c**: Competition between Δ*efg1* and WT cells. Open triangles, Ca-educated BALB/c mice treated with anti-CD4 antibody. Closed triangles, Ca-educated BALB/c mice treated with isotype control antibody. Panel **d**: Levels of *C. albicans* in fecal pellets collected on day 1, 6 or 18 post-inoculation from orally inoculated mice. Squares, WT *C. albicans*; circles, Δ*sin3* null mutant; triangles, Δ*sin3/SIN3* ^*+*^ complemented strain. Closed symbols, n-BALB/c mice; open symbols, BALB/c *nu/nu* mice

The results of these studies showed that in Ca-educated BALB/c mice, *Δefg1* cells were out-competed by both types of *EFG1*-expressing cells (Fig. 2a,b, closed black symbols); out-competition by the *Δefg1* strain was not observed at any time point measured. Total levels of colonization by either of the two mixes also declined over time (Additional file 3: Figure S2). Inoculation of n-BALB/c mice resulted in an initial out-competition by the *Δefg1* mutant relative to either type of *EFG1*-expressing strain, although *Δefg1* cells were eventually out-competed after ~9 days of colonization ([26]; summarized in Fig. 1b,c). In contrast, the *Δefg1* strain was able to out-compete both *ACT1pr-EFG1* cells (Fig. 2a, open symbols) and WT *C. albicans* cells (Fig. 2b, open symbols) in immunodeficient *nu/nu* BALB/c mice. Total levels of colonization were high in these mice (Additional file 3: Figure S2). When immunodeficient mice previously challenged with *C. albicans* (Ca-educated BALB/c mice *nu/nu* – not able to play strategy *a*) were inoculated with the mixture, the *Δefg1* null mutant strain out-competed WT cells (Fig. 2b, grey diamonds). This result contrasted with the results for Ca-educated BALB/c mice (constantly playing strategy *a*) inoculated with the same *C. albicans* mixture (Fig. 2b, closed triangles). In summary, the data showed that mixed populations of *Δefg1* and *EFG1*-expressing cells reached different final compositions depending on the immune status of the host.

**Fig. 3:**
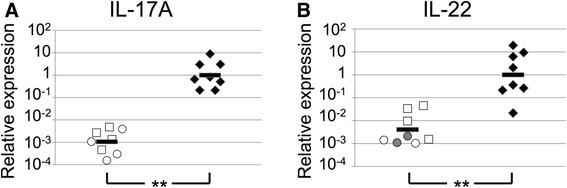
Expression of cytokines in GI tract tissue. Mice were orally inoculated with WT *C. albicans* and sacrificed 20 days later. Additional mice did not receive *C. albicans* inoculation and were sacrificed either on the day of inoculation (day 0) or on day 20. Expression of cytokines IL-17A (panel **a**) and IL-22 (panel **b**) in stomach tissue was measured by qRT-PCR and normalized using expression of GAPDH. Geometric mean for colonized mice was set to 1. Panels **a** and **b**: Circles, uninoculated, day 0; squares, uninoculated, day 20; filled diamonds, *C. albicans* colonized, day 20. Grey circles, samples with undetectable signal. Bar shows the geometric mean. **, *p <* 0.0001 (*t* test with log-transformed data)

The outcompetition by *EFG1*-expressing cells in Ca-educated BALB/c mice is proposed to reflect, at least partially, the activity of the host immune response. To show that immune responses were activated in response to *C. albicans* colonization, IL-17A and IL-22 cytokine expression in GI tract organs was measured. Cytokines in mice not colonized with *C. albicans* were also measured. These cytokines were chosen for analysis because previous studies showed that colonization resulted in up-regulation of their expression in the murine stomach [8, 51–53], the best studied site of interaction between *C. albicans* and GI tract tissue, as well as in other GI organs [8, 51]. We therefore extracted RNA from stomach tissue of mice colonized (or not) with *C. albicans* for 20 days and measured gene expression by real time RT-PCR (qRT-PCR). Uninoculated mice expressed relatively low levels of cytokine IL-17A, both at day 0 of the experiment (i.e., the day of inoculation, Fig. 3a open squares) and 20 days later (Fig. 3a, open circles). The same held true for cytokine IL-22 (Fig. 3b, open squares and circles respectively). Mice colonized with *C. albicans* for 20 days, the length of time used to generate Ca-educated BALB/c mice, expressed higher levels of IL-17A (Fig. 3a, closed diamonds) and IL-22 (Fig. 3b, closed diamonds). These results demonstrated that an immune response was mounted in *C. albicans-*colonized BALB/c mice*.*

Further, we proposed that CD4+ T cells would be a necessary part of a restrictive GI tract environment. The results of competitions in BALB/c *nu/nu* mice showed that T cells are required to generate a restrictive GI tract environment (Fig. 2b). To test for an ongoing role for CD4+ T cells, mice were first Ca-educated and then injected intraperitoneally with either anti-CD4 antibody, to deplete CD4+ T cells [46], or with isotype control antibody. The treated mice were orally inoculated with a mixture of *Δefg1* null and WT cells, and colonization by the two strains was monitored over time. The normal outcompetition by WT cells was not observed in Ca-educated mice treated with anti-CD4 antibody (Fig. 2c). Therefore, the results supported the model that the restrictive environment created by Ca-education was at least partially due to an immunological response. In summary, activity of the host immune system promotes colonization by fungal cells expressing *EFG1*, whereas an immunodeficient host favors *Δefg1* null cells.

### Parameter estimation

Equations 1 and 2 were used to simulate the behavior of *C. albicans* cells during GI colonization in mice. During the parameterization process, we postulated that in any host fungal fitness in response to a particular host strategy was the same.

To use the mathematical model to simulate colonization dynamics, we estimated values (in arbitrary units) for each of the eleven unknown parameters in the model: the four payoffs for the *C. albicans* cells, the four payoffs for the host cells, and three different values for ε describing rates of change from high to low Efg1p activity in each of the different hosts (*ε*_n ‐ BALB/c_, *ε*_*nu*/*nu*_ and *ε*_*Ca* − *educated*_: Eq. 1, Fig. 1a). Parameter estimation was conducted by fitting the model to the averaged experimental data shown in Fig. 1b,c or from our previous study [26] using the tool COPASI and its built-in Evolutionary Programming parameter estimation routine [54]. The parameter estimation results showed that different parameter sets successfully simulated the data. Therefore, we completed 1000 parameter estimation runs and visually inspected the model simulations for a number of fits. We noted that parameter sets with residual sum of squares (RSS) less than 0.07 approximated the data points well. Therefore, we performed further studies using parameter sets that gave RSS values smaller than 0.07 (393 total parameter sets). We plotted the distributions of fungal payoffs from the 393 parameter sets and, in most cases, did not observe large variations in fungal payoff estimates (Additional file 4: Figure S3A). We also performed correlation coefficient analysis for fungal payoffs. A high correlation in fungal payoff values was observed for fungi playing against a fixed host strategy (Additional file 4: Figure S3B). Using a representative set of parameters, we show that the model was able to simulate the experimental data (Fig. 1b,c).

### Estimated payoffs to fungal cells are in agreement with predictions based on experimental results

The relative values of the parameters in the payoff matrix (Fig. 1a) determine the overall dynamics of the competition between high and low Efg1p activity cells. Therefore, we examined how well the predicted relative values for payoffs to *C. albicans* corresponded to the experimentally known behavior of cells during colonization. The four fungal payoff values in a given parameter set were arranged from lowest to highest; this ordered sequence of payoff values is termed a motif. Each of the 393 parameter sets with RSS < 0.07 was analyzed and the observed frequencies for the various motifs are shown in Table 1. Although the values for parameters in different parameter sets were not identical, two parameter orders accounted for over 90 % of the parameter sets that successfully simulated the data. Therefore, most of the successful parameter sets exhibited similar features.

**Table 1:**
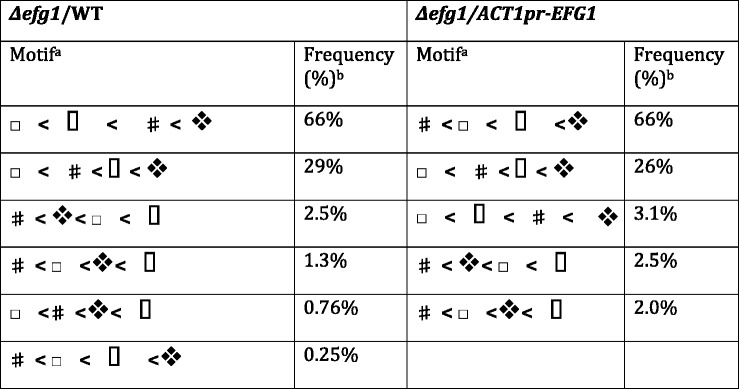
Fungal parameter motifs

^a^Description of fungal payoff motifs. , *C. albicans* low *EFG1,* host basal; , *C. albicans* high *EFG1*, host basal; , *C. albicans* high *EFG1*, host activated; , *C. albicans* low *EFG1*, host activated

^b^The frequency of each type of parameter order

The symbol  will be used to indicate the payoff to *C. albicans* when the fungus is expressing low levels of *EFG1* and the host is using the basal strategy.  will be used to indicate the fungal payoff when *C. albicans* is expressing *EFG1* highly and the host is using the basal strategy. When the host is using the activated strategy, *C. albicans* expressing *EFG1* at low levels will receive payoff  and *C. albicans* expressing *EFG1* highly will receive payoff .

For the *C. albicans* population composed of *Δefg1* and WT cells, 6 motifs were observed and the two most common were  (frequency 66 %) and  (frequency 29 %) (Table 1 and Fig. 1a). The structures of these motifs agreed well with experimental expectations (Fig. 2 and [26, 30]). Based on experimental results, the greatest payoff for *C. albicans* (allowing highest colonization) should occur in either of the following two situations. In the first situation, *C. albicans* cells with low *EFG1* expression (*l*) colonize a naïve host (strategy *b*) to high levels (Fig. 2, open symbols), presumably because the host immune response is too weak to kill the fungus, resulting in high payoff  to *C. albicans*. In the second situation, *C. albicans* cells in the high Efg1p activity state (*h*) resist killing in a host with a stronger immune response (strategy *a*) and colonize well (Fig. 2, closed symbols), resulting in high payoff *.* In either situation, *C. albicans* is well adapted to the host environment. Therefore, in a host with an activated immune response, the payoff should be greater for a *C. albicans* cell with high Efg1p activity () than one with low Efg1p activity *,* while in a naïve host, low Efg1p activity should give a higher payoff () than high Efg1p activity , and indeed, this is observed in all six motifs (Table 1). Further, in the top two motifs for each competition, colonization of the naïve host by cells with low Efg1p activity state, (), results in the largest payoff value, arguing that the situation is maximally advantageous to *C. albicans* when the host is immunocompromised.

Additionally, in the two most common motifs,  is larger than , i.e., cells with high Efg1p activity in a naïve host are better off than cells with low Efg1p activity that are colonizing a host with a stronger immune response. This prediction agrees well with experimental results because when the host has mounted an activated immune response, low Efg1p activity cells may be preferentially killed (Fig. 1b,c, green and blue line). Moreover, the smaller payoff  implies that there is a larger penalty associated with having the low Efg1p activity state (*l*) when the host is using the activated immune response (*a*), compared to the penalty for having the high Efg1p state (*h*) when the host is using a naïve immune response (*b*).

For the *Δefg1/ACT1pr-EFG1* population, the payoff values were classified into five different motifs (Table 1) and the two most common were:

(frequency 66 %) and  (frequency 26 %). As in the *Δefg1*/WT competition, it holds true that  in all five motifs. Again, in terms of fungal payoffs, this inequality implies that in a host with an activated immune response, *C. albicans* cells with high Efg1p activity are more fit, whilst in a naïve host, cells with low Efg1p activity are favored. Payoff to fungal cells with high Efg1p activity in n-BALB/c mice (using strategy *b*) was relatively small for the *Δefg1*/*ACT1pr-EFG1* competition in comparison to the *Δefg1*/WT competition. This difference in the payoff is consistent with the finding that at early timepoints post-inoculation of n-BALB/c mice, the *ACT1pr-EFG1* strain colonizes poorly relative to WT cells [26]. If we omit this payoff (), the most common motifs for the two competitions are identical. Thus, when the model successfully simulated the experimental data (RSS < 0.07), the relative values in the payoff matrix showed good correspondence with the experimentally determined behavior of *C. albicans* cells.

### The rate at which *C. albicans* changes from high to low Efg1p activity states is predicted to be greater in a host with a weak immune response

Equation 1 includes a term *ε(*1 *- x*_*l*_*)* that takes into account the effect of changes in Efg1p activity that may occur in individual WT *C. albicans* cells. To detect the contribution of changes in Efg1p activity in individual cells to colonization dynamics, we repeated the parameter estimation using data for competitions in individual mice (as depicted in Fig. 2b). Using this approach, we asked how *ε* values varied between mice as a result of their individual immune responses. To determine values for *ε* we fitted parameters for the *Δefg1*/WT competition for each mouse individually (four Ca-educated mice, three BALB/c *nu/nu* mice, two Ca-educated BALB/c *nu/nu* mice and four n-BALB/c mice [26]). Each parameter set that simulated the *Δefg1*/WT competition dynamics for an individual mouse and also simulated the averaged data (shown in Fig. 1c) was considered successful. Next, the median RSS value for each mouse was used as the threshold for accepting parameter sets for further analysis. From these accepted parameter sets, we extracted values for *ε,* the rate at which cells change from high Efg1p activity to low Efg1p activity. The *ε* values were then averaged and plotted in Fig. 1d. Including a term for the converse event, changing from low Efg1p activity to high, did not improve the model fit and this event was ignored. This event may be negligible because at the start of the experiment, few WT cells were in the low *EFG1* state [26]).

In Ca-educated mice, the predicted rate at which cells changed from high to low Efg1p activity, *ε*_Ca-educated_, was relatively low (Fig. 1d). In contrast, in n-BALB/c mice, the rate *ε*_n-BALB/c_ (averaged over the 18-day time course) was higher (Fig. 1d, *p <* 0.027, *t*-test with log-transformed data). The highest rate of change, *ε*_*nu/nu*_, was predicted to occur during colonization of BALB/c mice *nu/nu* (Fig. 1d; *p <* 0.019 vs n-BALB/c mice and *p <* 0.0037 vs Ca-educated mice; *t*-test with log-transformed data). Therefore, we observed an inverse relationship between the strength of the host immune response and *ε*.

We also tested the effects of setting *ε* to either zero or a non-zero constant value. Using simulation, we found that the fit of the model to the experimental data was poor under these conditions (Additional file 5: Figure S4). Therefore, the modeling predicted that the rate at which WT cells alter *EFG1* expression was not constant; rather, the rate of change differed depending on host immune status.

### Regulators of *EFG1* expression are differentially expressed based on host immune status

**Fig. 4:**
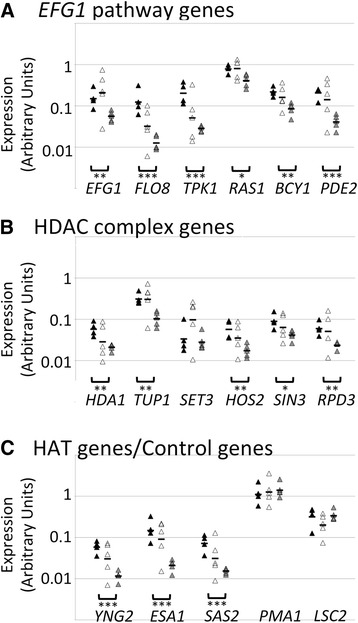
Expression of genes in *C. albicans* cells colonizing mice of different immune status. Ca-educated (black symbols), n-BALB/c mice (open symbols) and BALB/c mice *nu/nu* (grey symbols) were orally inoculated with *C. albicans*. On day 2 post-inoculation, *C. albicans* cells were harvested and gene expression in these cells was measured by quantitative RT-PCR. The experiment was performed twice and representative results are shown. Brackets indicate comparisons between expression in Ca-educated mice versus BALB/c *nu/nu* mice. *, *p <* 0.05; **p < 0.01; ***p < 0.001 (*t* test with log-transformed data). Expression of the following genes is shown: Panel **a**: *EFG1*, *FLO8, TPK1, RAS1, BCY1* and *PDE2*. Panel **b**: *HDA1, TUP1, SET3, HOS2, SIN3,* and *RPD3*. Panel **c**: *YNG2, ESA1*, *SAS2*, *PMA1* and *LSC2*

Our modeling results support the idea that individual *C. albicans* cells change from high Efg1p activity to low Efg1p activity at different rates depending on the GI tract environment. We therefore hypothesize that changes in the environment are sensed, resulting in altered gene expression. To further validate this point, we measured the expression of factors that regulate Efg1p. RNA from WT *C. albicans* cells harvested from the ceca of Ca-educated, n-BALB/c, and BALB/c *nu/nu* mice on day 2 post-inoculation was extracted, and qRT-PCR was performed. As before [26], expression of *EFG1* was lower in *C. albicans* cells recovered from BALB/c *nu/nu* versus Ca-educated BALB/c mice (Fig. 4a). At this early time point, *EFG1* expression in n-BALB/c mice was variable. We next studied expression of a collection of genes whose products regulate the activity of Efg1p. *TPK1, BCY1, RAS1, PDE2* [55, 56] and *FLO8*, whose product functions with Efg1p [57], showed lower expression in the immunodeficient host (Fig. 4a). *RAS1* (R^2^ = 0.63) and *PDE2* (R^2^ = 0.7) expression correlated well with *EFG1* expression (Additional file 6: Figure S5).

*EFG1* expression is also influenced by activities of several histone modification complexes [58–60]. Therefore, we determined the expression levels of these genes in *C. albicans* cells derived from colonized mice with varying immune status. Consistent with the determined rates of change for *EFG1* expression in response to the GI tract environment (Fig. 1d), we observed a trend in the expression levels of *EFG1* regulators depending on host immune status. *HDA1, SET3, HOS2, SIN3,* and *RPD3* encode components of histone deacetylase complexes (HDAC) and they are known to regulate the expression of *EFG1* [58–60]. Transcript levels for these genes and for the co-repressor gene *TUP1* were 2–3-fold lower in *C. albicans* cells colonizing BALB/c *nu/nu* mice in comparison with cells colonizing Ca-educated mice (Fig. 4b). *TUP1* (R^2^ = 0.72) and *RPD3* (R^2^ = 0.71) expression correlated well with *EFG1* expression (Additional file 6: Figure S5). A classical role of HDAC proteins is transcriptional repression as a result of more closed and compact chromatin state [61]. In contrast to HDACs, histone acetyltransferases (HATs) are commonly found to enhance transcription [61]. For example, Efg1p recruits some HATs to promoters of hyphae-specific genes, up-regulating their expression [62]. Expression of HAT genes was also regulated in a host status-dependent manner. HAT genes *ESA1* and *SAS2* [62, 63] were expressed at 5 to 7-fold lower levels during colonization of BALB/c *nu/nu* mice (Fig. 4c), conditions in which *EFG1* expression decreased from high to low at a faster rate (Fig. 1d). *ESA1* (R^2^ = 0.68) and *YNG2* (R^2^ = 0.59) expression correlated well with *EFG1* expression (Additional file 6: Figure S5). These observations suggest that regulation of gene expression through the function of HAT and HDAC complexes plays a role in finding optimal strategies during host-pathogen interactions.

Finally, as expected, housekeeping genes *PMA1,* and *LSC2* were expressed at similar levels in cells colonizing different hosts (Fig. 4c). Therefore, in addition to Efg1p, several proteins that function in the Efg1p pathway showed differential expression depending on host immune status.

### Altered colonization dynamics in the absence of HDAC subunit Sin3p

These results indicated that histone modifying activities might play a role in modulating colonization. To test this model, we constructed and studied mutants lacking *SIN3,* encoding a subunit of a histone deacetylase complex. Both *SIN3* and *RPD3,* encoding the catalytic subunit of the Sin3p complex, showed host status dependent differences in gene expression (Fig. 4). Further, *EFG1* expression was lower during exponential growth of *C. albicans* cells in the absence of Rpd3p [59]. Therefore, we constructed a homozygous *Δsin3* null mutant in which both copies of *SIN3* were deleted, and then complemented the mutation by reintroducing one copy of *SIN3* into the null mutant at its native locus.

To measure the effects of the Δ*sin3* mutation on colonization, n-BALB/c and BALB/c *nu/nu* mice were colonized with WT, Δ*sin3* null, or the complemented strain. Overall, as previously observed [26, 44], colonization was higher in mice lacking T cells for all strains when compared to the levels in a WT host. At day one post-inoculation, there was no difference in colonization between WT and the Δ*sin3* strain in either a healthy or a T cell deficient host (Fig. 2d). Δ*sin3* cells showed higher levels of colonization at day 6 post-inoculation, relative to WT *C. albicans,* in BALB/c *nu/nu* mice (Fig. 2d; *p <* 0.012). This phenotype was reversed by complementation. Hyper-colonization by the Δ*sin3* mutant strain was not observed in n-BALB/c mice. These observations showed that the Δ*sin3* mutant strain achieved high colonization in an immunodeficient mouse more rapidly than WT *C. albicans*, consistent with the model that modulation of gene expression through histone modifying activities affects colonization dynamics. Differences in *EFG1* expression were not detected (data not shown) but subtle or transient differences would be difficult to detect due to the variability in *C. albicans* gene expression in different mice. We conclude that Sin3p affects the dynamics of *C. albicans* colonization in the GI tract.

## Discussion

Central to the biology of *C. albicans* is its ability to colonize a healthy host as a commensal and to become an invasive pathogen in a compromised host. *C. albicans* populations colonizing the GI tracts of immunocompetent or immunodeficient hosts differ in expression of *EFG1* (Fig. 4 and [26]). Here we argue that two mechanisms are important for shaping the fungal population in response to host immune status. One, selective forces from the host act on the fungal population. In this mechanism, fungi are passively acted on. Two, fungi respond to host immune status by altering gene expression. In this mechanism, fungi actively sense and respond to host immune status accordingly. The combined effect of both mechanisms produces the observed host status dependent difference in fungal populations.

The results of this study support the conclusion that the rate of change from high to low Efg1p activity is higher in an immunodeficient or naïve host. Production of cells with low Efg1p activity in a host with a strong immune response should be evolutionarily costly because these cells could be killed. Controlling the rate of change in Efg1p activity protects against this evolutionary cost by minimizing the number of maladapted cells produced. Thus, by regulating the rate at which cells change from high to low Efg1p activity, the fittest *C. albicans* population is produced for each type of host.

Higher expression of HAT and HDAC genes such as *RPD3*, which is required for higher *EFG1* expression in *C. albicans* cells [59], may result in higher expression of *EFG1* during colonization. Consistent with this model, deletion of an HDAC subunit resulted in altered colonization dynamics. The effects of the Δ*sin3* null mutation on colonization could reflect transient differences in expression of *EFG1* between WT and mutant cells. In particular, *EFG1* expression may reach low levels sooner in *Δsin3* null mutants during colonization of an immunodeficient host, resulting in a more rapid rise in colonization. Alternatively, since HDACs are expected to affect the expression of many genes, a number of genes may exhibit altered expression in the absence of this HDAC subunit and the sum of their effects may yield the altered colonization that was observed. These results are consistent with the model that there is a profound difference in the physiology of *C. albicans* cells colonizing an immune active, immunocompetent host versus an immunodeficient host.

Most humans are naturally colonized with *Candida* early in life [64] and would be expected to exhibit an “activated” response to *C. albicans.* Therefore, *C. albicans* populations colonizing healthy humans are expected to express high levels of *EFG1* transcripts*.* If a person’s immune status were to decline, the *C. albicans* population would be expected to shift so that cells express low levels of *EFG1* transcripts. A change in the population resulting in a preponderance of low Efg1p activity cells may represent an early step in candidiasis. Cells lacking Efg1p were previously found to escape more readily from the GI tract in mice subjected to hypoxic shock [65]. Therefore, for WT cells, low *EFG1* expression may favor escape from the GI tract and after escape, *EFG1* expression may increase, permitting expression of Efg1p-dependent virulence factors and favoring the progression to disease.

In summary, our results using a mathematical model based on evolutionary game theory support the conclusion that the ability of cells to sense the immune status of the host and down-regulate Efg1p activity accordingly contributes to the adaptation of the population to the host. Evolutionary game theory allowed the analysis of the complex interactions between host and *C. albicans* cells as they changed in response to each other during the course of colonization. Since host status may decline as a result of numerous insults to the host, increasing the rate of change from high to low Efg1p activity under appropriate conditions enhances the ability of *C. albicans* to colonize to high level. Through differential regulation of fungal activities such as Efg1p, the physiology of the colonizing fungal population can be tightly coupled to the immune status of the host.

## Conclusion

In conclusion, here we present an application of evolutionary game theory to study mechanisms important for *C. albicans* colonization of hosts with varying immune activities. Our modeling approach supported the idea that *C. albicans* senses host immune strength and adapts accordingly. Our mathematical model predicted that individual fungal cells were able to decrease Efg1p activity at a faster rate when colonizing an immunodeficient host. We showed that in response to host immune status, *C. albicans* regulates both up- and down-stream *EFG1*-related processes by altering expression of multiple histone modification enzymes involved in modulating the overall chromatin structure in the fungal cell. Taken together, our results demonstrate that *C. albicans* cells fine-tune their regulatory programs, including the Efg1p pathway, in response to host immune activities.

## Methods

### Strains and growth conditions

*C. albicans* strains used in this study are listed in (Additional file 7: Table S1). Primers used for strain construction are listed in (Additional file 8: Table S2B).

The Δ*sin3* null mutant and complemented strains were constructed using the strategy described previously [66]. Briefly, primer pairs SIN3A/SIN3B and SIN3C/SIN3D were used to amplify sequences from the 5′ and 3′ regions of the *SIN3* gene. These fragments were fused by overlap PCR and cloned into either the Sat placer or the Ura placer [66]. The two constructs were used to delete the two alleles of *SIN3* from the chromosome of a WT strain. Integration of the constructs was verified by PCR with primers Sn3S3F/Sn3S3R for the Sat placer construct and primers Sn3U5F/Sn3U5R for the Ura placer construct. Deletion of the ORF was verified by PCR with primers Sn3OF/Sn3OR. For complementation, the *SIN3*^*+*^ gene was amplified with primers Sin3cF/Sin3cR and cloned into the Ura placer [66].

YPD (1 % yeast extract, 2 % peptone, 2 % glucose) and minimal CM media lacking uracil were as described [67]. Mouse inocula were grown at 37 °C in YPD for 24 h.

### GI colonization in mice

All experiments were done in compliance with Tufts University IACUC guidelines. 5–7 week old female BALB/c or BALB/c *nu/nu* mice (NCI) were treated with tetracycline 1 mg/ml, streptomycin 2 mg/ml, gentamicin 0.1 mg/ml [26]. Mice were tested for fungal contamination prior to inoculation and inoculated with 5x10^7^*C. albicans* cells by oral gavage. Colonization was measured in fecal pellets collected at 4 h, 8 h, 24 h, 30 h, 48 h, 3 days and 6 days post-inoculation by homogenizing and plating [26]. The fraction of *Δefg1* null mutant cells was defined as: #*Δefg1*/(#*Δefg1* + #(*ACT1pr-EFG1* or WT)).

For Ca-educated mice, mice treated with antibiotics as above were inoculated by oral gavage with *C. albicans* strain SN100 (His^−^; a WT, genetically marked strain). Twenty days post-inoculation, antibiotics were replaced with fluconazole (60 or 120 ug/ml) in drinking water. Following fluconazole treatment SN100 colonization declined 100–1000 fold. After 3 days, fluconazole was replaced by antibiotics (tetracycline, streptomycin, gentamicin) and 3 days later, mice were inoculated by oral gavage with mixtures of *Δefg1* null mutant cells and *EFG1-*expressing cells. Colonies were counted [26] and the fraction of *Δefg1* null mutant cells was defined as: #*Δefg1*/(#*Δefg1* + #(*ACT1pr-EFG1* or WT) + #SN100). The majority of the cells were derived from the second gavage. Experiments were repeated at least twice.

In some experiments, Ca-educated BALB/c mice were injected with 200 μl anti-CD4 antibody (clone GK1.5, 200 μg, Biolegend) or 200 μl isotype control (Rat IgG2b, 200 μg, Biolegend) intraperitoneally. Injections were given two days before and on the same day as inoculation with the mixture of two strains of *C. albicans,* as described previously [46].

Experimental data from a representative experiment with 2–3 mice per competition were averaged and used for fitting parameters. For modeling the *Δefg1*/WT cell population, the proportion of low Efg1p activity cells was estimated by measuring the fraction of *Δefg1* cells, since the number of WT cells with very low Efg1p activity is initially small [26].

### Measurement of gene expression by quantitative RT-PCR

Ca-educated, n-BALB/c or BALB/c *nu/nu* mice (4–5 per group) were inoculated with *C. albicans* by gavage*.* On day 2 post-inoculation, mice were euthanized and the contents of the ceca were collected [26]. Gene expression was analyzed as described [26] except that after normalization with *ACT1*, expression is presented in arbitrary units. Primers used are listed in (Additional file 8: Table S2A ).

For measurement of cytokine gene expression, stomach tissue from *C. albicans*-colonized or uncolonized mice was frozen at −80 °C in RNALater. RNA was purified with Trizol extraction and column purification, using the Ambion Purelink mini kit. DNA was eliminated with on-column DNase treatment. cDNA preparation with Superscript III and analysis of gene expression was as described [26] except that expression of GAPDH was used for normalization. Previously described primers, listed in (Additional file 8: Table S2A ), were used [68–70].

## Additional files

Additional file 1: Figure S1. Illustration of *C. albicans*-host cell interactions. Panel A: Fungal cells are depicted as ovals. Open oval, low Efg1p activity state (*l*); closed oval, high Efg1p activity state (*h*). Host cells are depicted as rectangles. Open rectangle, basal state (*b*); closed rectangle, activated state (*a*). Panel B: populations of *C. albicans* cells (ovals) interacting with populations of host cells (rectangles). Letters indicate various conditions and arrows indicate changes in populations that occur over time. Condition A indicates a fungal population in which half of the cells have low Efg1p activity (open ovals) and half have high Efg1p activity (filled ovals). The host cells in this condition are activated (filled rectangles). This condition represents interactions occurring following inoculation of a mixed population of *C. albicans* cells into a Ca-educated host. As shown by the data in Fig. 2, the fungal population changes over time so that almost all of the cells have high Efg1p activity as illustrated by Condition B. Condition C depicts the situation following inoculation of the same fungal population into a T cell deficient BALB/c *nu/nu* mouse. Due to immunodeficiency, activated host cells are not produced in the normal way during colonization (open rectangles) and the fungal population changes so that most of the cells have low Efg1p activity (depicted by Condition D). Panel C: populations of *C. albicans* cells interacting with populations of host cells are depicted. Letters indicate various conditions and arrows indicate changes in populations that occur over time. Condition X depicts the inoculation of a mixed fungal population into an initially naïve n-BALB/c mouse. Initially, the low Efg1p activity cells out-compete resulting in the population shown in Condition Y. However, as host cells change to the activated state, the fungal population changes, resulting in Condition Z. (TIF 1067 kb) Additional file 2:
**Derivation of equations used for modeling.** (PDF 1657 kb) Additional file 3: Figure S2. Colonization over time in Ca-educated mice and BALB/c *nu/nu* mice. For the mice whose colonization is depicted in Fig. 2a and b, the total CFU/gm fecal pellets was calculated. CFU/gm is shown for fecal pellets collected on Day 1, Day 2, and Day 6 post-inoculation with the mixture of 2 *C. albicans* strains. Each symbol shows the results from an individual mouse and the bar shows the geometric mean. Black triangle, BALB/c Ca-educated mice. Open diamond, BALB/c *nu/nu* mice. (TIF 244 kb) Additional file 4: Figure S3. Parameter analysis. Panel A: Distribution of fungal payoffs. In a host playing the activated strategy, *a*, the *efg1* null strain typically had the lowest payoff. In a host playing the basal strategy, *b*, the *ACT1pr-EFG1* strain received the lowest payoff. The payoff to WT cells in the naïve or immunodeficient host was most variable. Panel B: Correlation analysis of fungal payoffs. Values for fungal payoff were highly correlated when fungi were playing against a fixed host strategy. Fungal payoffs in one host environment did not correlate with payoffs in another host environment. (TIF 433 kb) Additional file 5: Figure S4. Simulation of experimental data when values for *ε* were changed. To determine how much values for *ε* influenced colonization dynamics, the value of *ε* in all hosts was set to either 0 (Panel A) or to the value estimated for n-BALB/c mice (Panel B). The model was then used to simulate *C. albicans* colonization dynamics. Orange, BALB/c *nu/nu* mice; blue, n-BALB/c mice; green, Ca-educated mice. When *ε* was set to zero, model simulations predicted the Δ*efg1* null mutant strain to be completely outcompeted by WT cells in n-BALB/c mice within 6 days post inoculation. When *ε* was set to the value observed in n-BALB/c mice, the resulting model simulations predicted that the Δ*efg1* null mutant strain would persist in Ca-educated mice. Thus, when the value of *ε* was changed, a poor fit of the model to the data was observed. (TIF 112 kb) Additional file 6: Figure S5. Correlation between transcript levels of *EFG1* and other genes of interest. Normalized expression levels for genes shown in Fig. 4 are plotted as a function of *EFG1* expression (shown on the x-axis in all panels). Each symbol indicates expression in *C. albicans* cells from an individual mouse. Expression of several genes correlated well with the expression of *EFG1. (TIF 511 kb)*
Additional file 7: Table S1.
*C. albicans* strains used in this study. (PDF 54 kb) Additional file 8: Table S2. Primers used in this study. (PDF 50 kb)
