# Supplementary figures and images for: The game theory of Candida albicans colonization dynamics reveals host status-responsive gene expression
Source: BMC Syst Biol. 2016 Mar 1;10:20. doi: 10.1186/s12918-016-0268-1 (PMC4772284; doi:10.1186/s12918-016-0268-1)

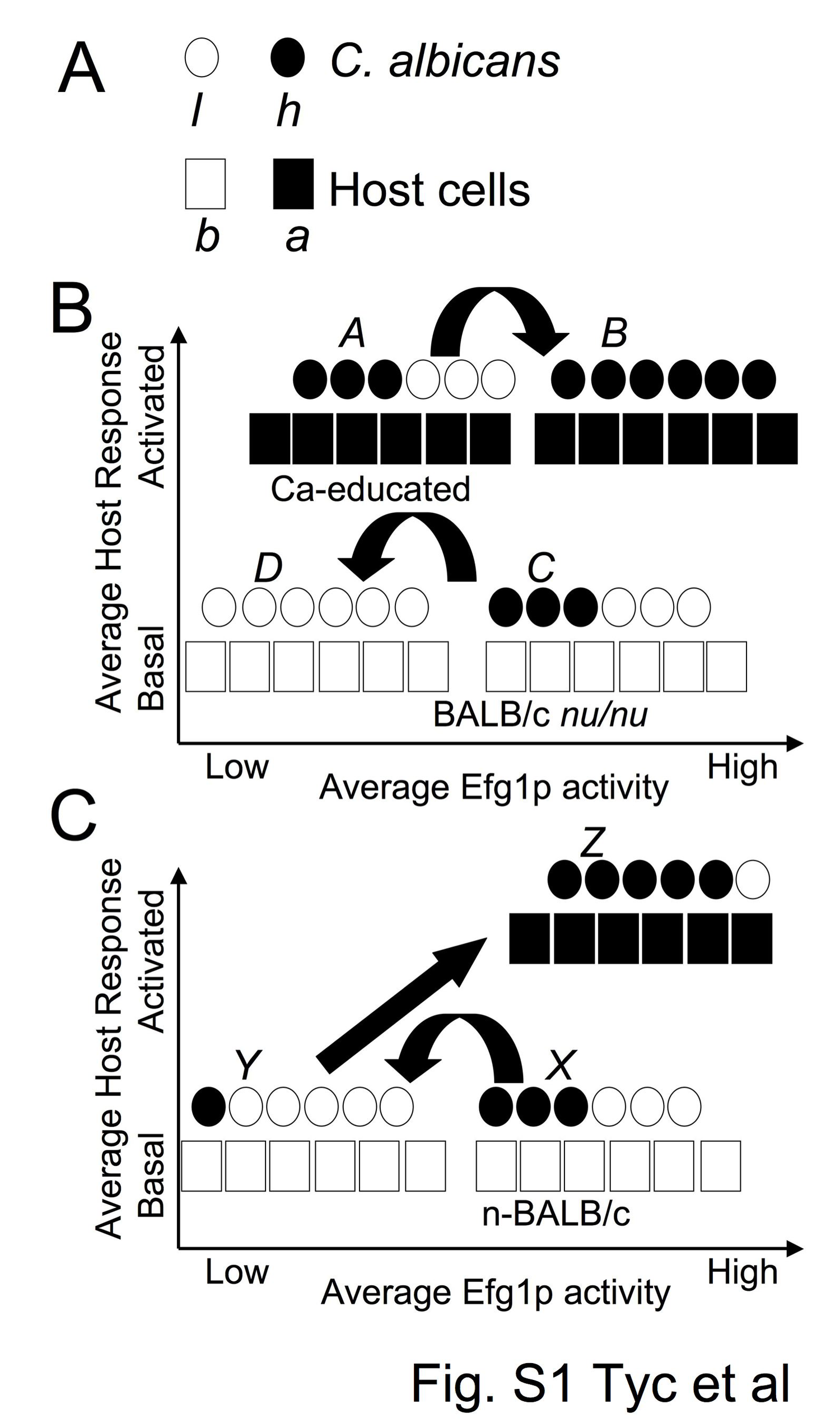

Supplement: Additional file 1: Figure S1. — Illustration of C. albicans-host cell interactions. Panel A: Fungal cells are depicted as ovals. Open oval, low Efg1p activity state (l); closed oval, high Efg1p activity state (h). Host cells are depicted as rectangles. Open rectangle, basal state (b); closed rectangle, activated state (a). Panel B: populations of C. albicans cells (ovals) interacting with populations of host cells (rectangles). Letters indicate various conditions and arrows indicate changes in populations that occur over time. Condition A indicates a fungal population in which half of the cells have low Efg1p activity (open ovals) and half have high Efg1p activity (filled ovals). The host cells in this condition are activated (filled rectangles). This condition represents interactions occurring following inoculation of a mixed population of C. albicans cells into a Ca-educated host. As shown by the data in Fig. 2, the fungal population changes over time so that almost all of the cells have high Efg1p activity as illustrated by Condition B. Condition C depicts the situation following inoculation of the same fungal population into a T cell deficient BALB/c nu/nu mouse. Due to immunodeficiency, activated host cells are not produced in the normal way during colonization (open rectangles) and the fungal population changes so that most of the cells have low Efg1p activity (depicted by Condition D). Panel C: populations of C. albicans cells interacting with populations of host cells are depicted. Letters indicate various conditions and arrows indicate changes in populations that occur over time. Condition X depicts the inoculation of a mixed fungal population into an initially naïve n-BALB/c mouse. Initially, the low Efg1p activity cells out-compete resulting in the population shown in Condition Y. However, as host cells change to the activated state, the fungal population changes, resulting in Condition Z. (TIF 1067 kb) [file 12918_2016_268_MOESM1_ESM.tif]

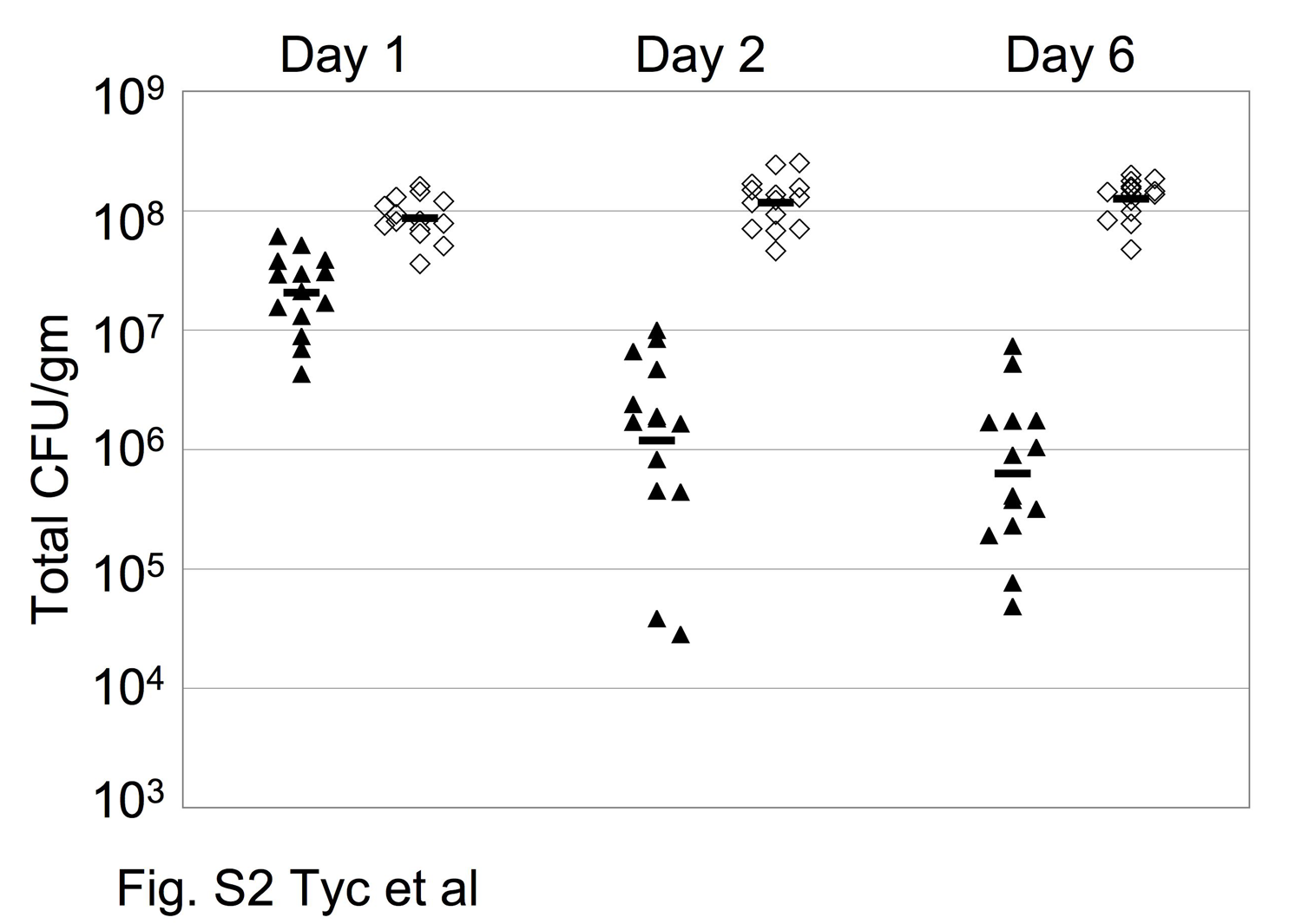

Supplement: Additional file 3: Figure S2. — Colonization over time in Ca-educated mice and BALB/c nu/nu mice. For the mice whose colonization is depicted in Fig. 2a and b, the total CFU/gm fecal pellets was calculated. CFU/gm is shown for fecal pellets collected on Day 1, Day 2, and Day 6 post-inoculation with the mixture of 2 C. albicans strains. Each symbol shows the results from an individual mouse and the bar shows the geometric mean. Black triangle, BALB/c Ca-educated mice. Open diamond, BALB/c nu/nu mice. (TIF 244 kb) [file 12918_2016_268_MOESM3_ESM.tif]

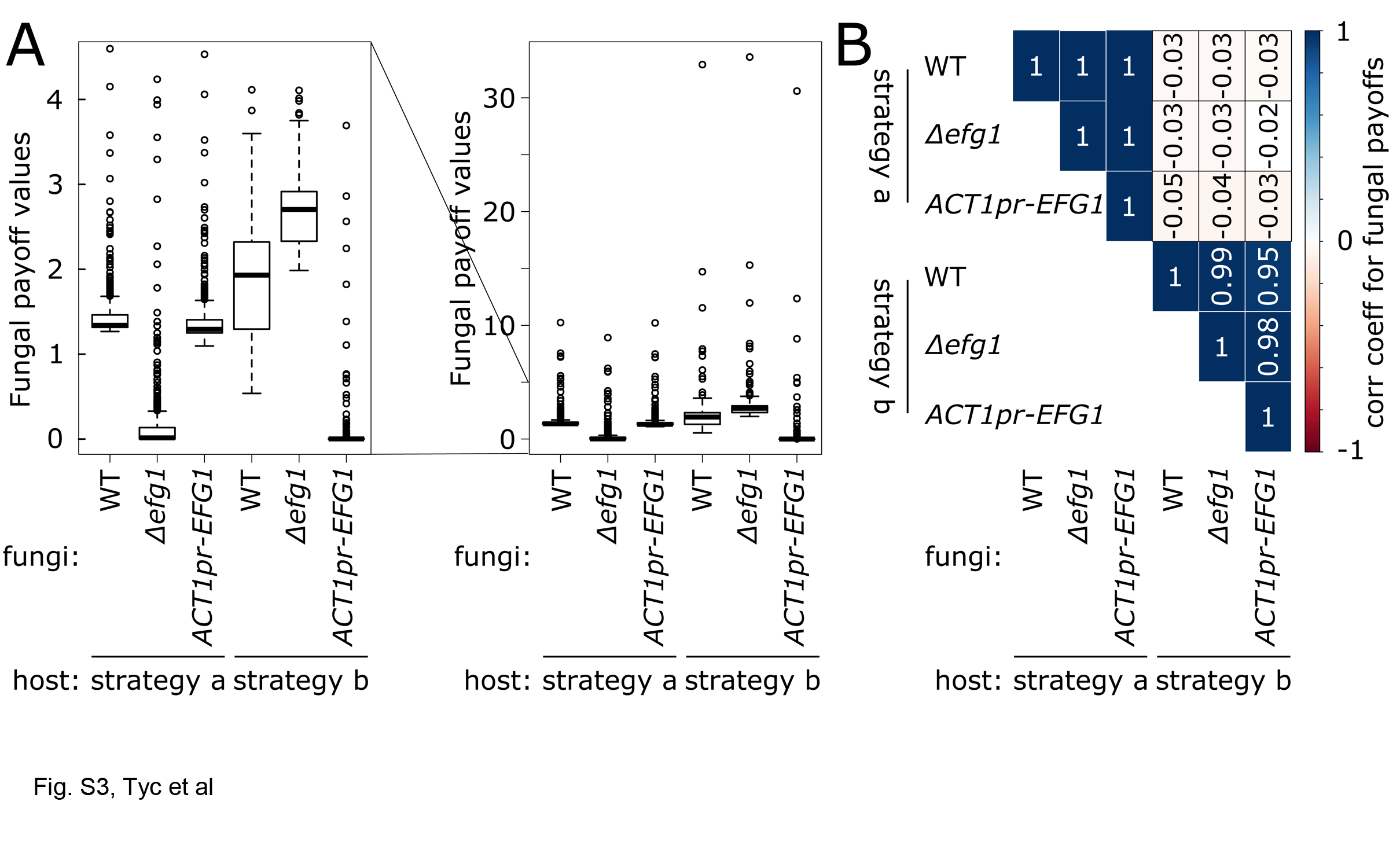

Supplement: Additional file 4: Figure S3. — Parameter analysis. Panel A: Distribution of fungal payoffs. In a host playing the activated strategy, a, the efg1 null strain typically had the lowest payoff. In a host playing the basal strategy, b, the ACT1pr-EFG1 strain received the lowest payoff. The payoff to WT cells in the naïve or immunodeficient host was most variable. Panel B: Correlation analysis of fungal payoffs. Values for fungal payoff were highly correlated when fungi were playing against a fixed host strategy. Fungal payoffs in one host environment did not correlate with payoffs in another host environment. (TIF 433 kb) [file 12918_2016_268_MOESM4_ESM.tif]

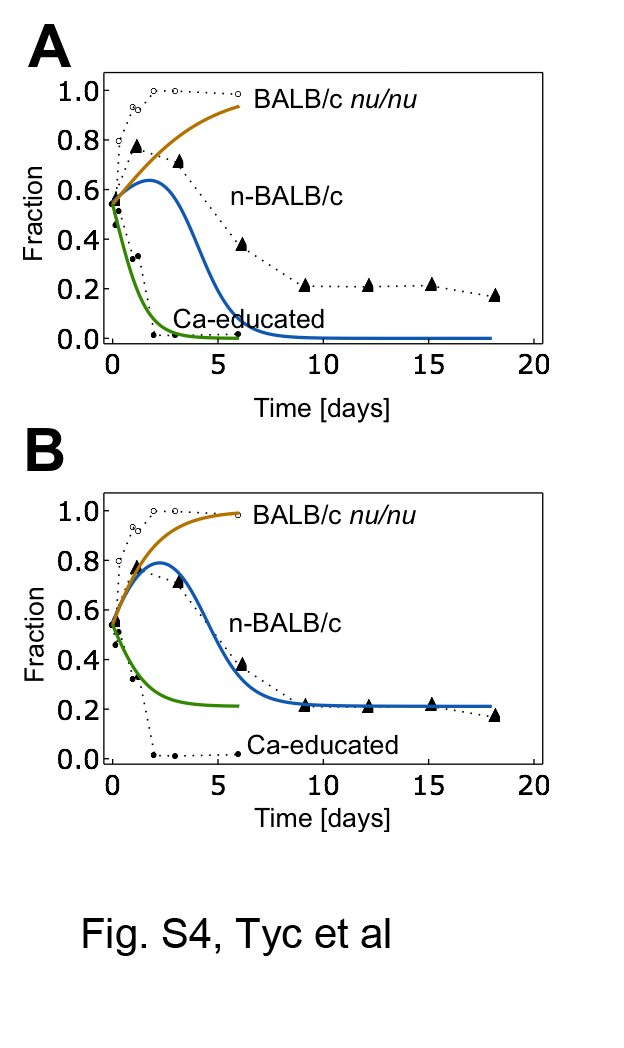

Supplement: Additional file 5: Figure S4. — Simulation of experimental data when values for ε were changed. To determine how much values for ε influenced colonization dynamics, the value of ε in all hosts was set to either 0 (Panel A) or to the value estimated for n-BALB/c mice (Panel B). The model was then used to simulate C. albicans colonization dynamics. Orange, BALB/c nu/nu mice; blue, n-BALB/c mice; green, Ca-educated mice. When ε was set to zero, model simulations predicted the Δefg1 null mutant strain to be completely outcompeted by WT cells in n-BALB/c mice within 6 days post inoculation. When ε was set to the value observed in n-BALB/c mice, the resulting model simulations predicted that the Δefg1 null mutant strain would persist in Ca-educated mice. Thus, when the value of ε was changed, a poor fit of the model to the data was observed. (TIF 112 kb) [file 12918_2016_268_MOESM5_ESM.tif]

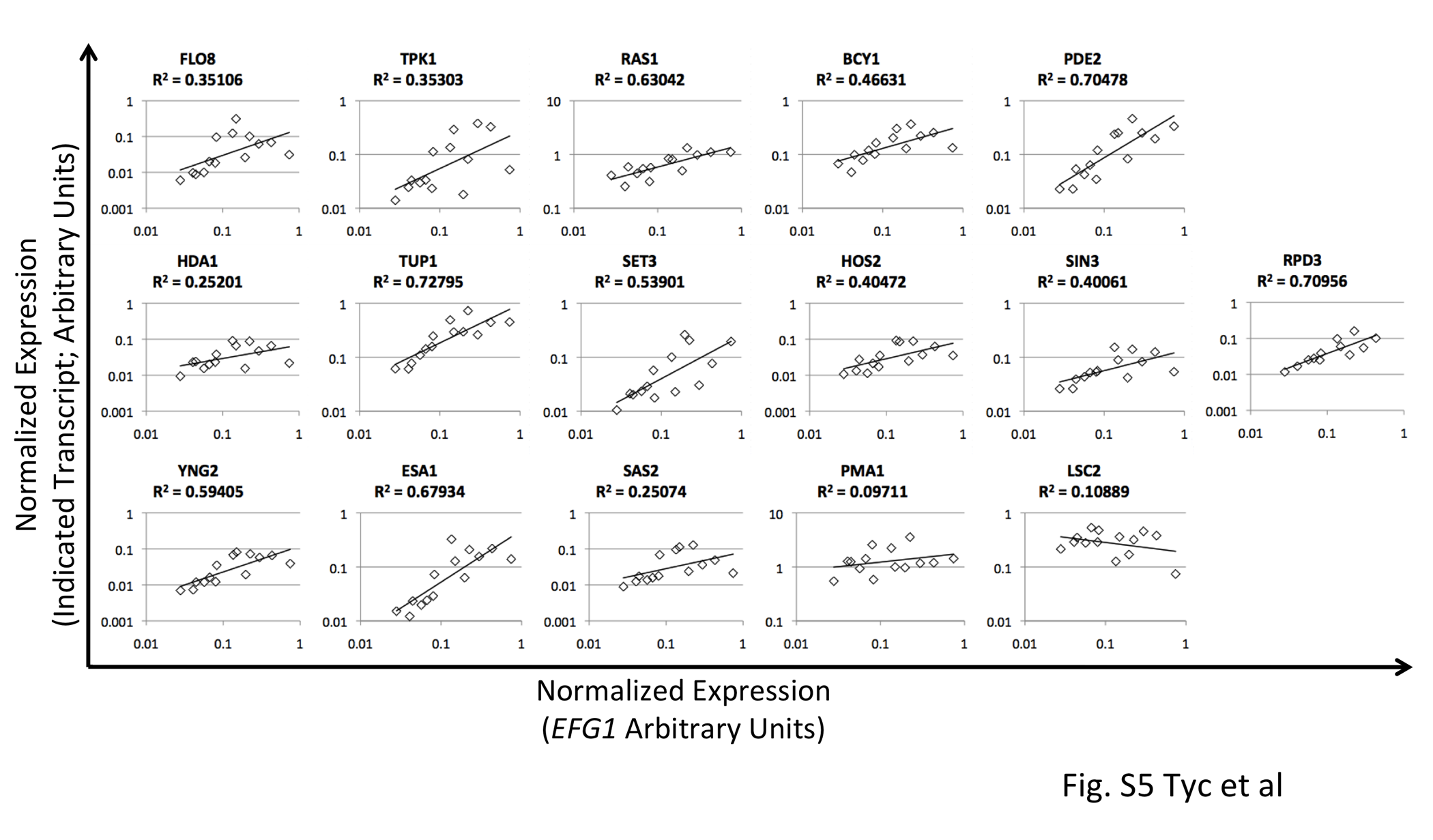

Supplement: Additional file 6: Figure S5. — Correlation between transcript levels of EFG1 and other genes of interest. Normalized expression levels for genes shown in Fig. 4 are plotted as a function of EFG1 expression (shown on the x-axis in all panels). Each symbol indicates expression in C. albicans cells from an individual mouse. Expression of several genes correlated well with the expression of EFG1. (TIF 511 kb) [file 12918_2016_268_MOESM6_ESM.tif]
