# Supplementary material for: The game theory of Candida albicans colonization dynamics reveals host status-responsive gene expression
Source: BMC Syst Biol. 2016 Mar 1;10:20. doi: 10.1186/s12918-016-0268-1 (PMC4772284; doi:10.1186/s12918-016-0268-1)
Supplement: Additional file 2: — Derivation of equations used for modeling. (PDF 1657 kb) [file 12918_2016_268_MOESM2_ESM.pdf]

**Equations**

We first defined $n_l$ to be the number of *C. albicans* cells with low Efg1p activity and $n_h$ to be the number of cells with high Efg1p activity. The total number of cells in the fungal population, $N$, is:

$$N = n_l + n_h \qquad \text{Eq. S1}$$

The proportion of either $n_l$ or $n_h$ cells in the population is defined as:

$$x_i = \frac{n_i}{N} \qquad \text{Eq. S2}$$

where $i$ denotes either fungal strategy, $l$ or $h$.

Similarly for the host, the number of host immune cells using the naïve strategy is $k_b$ and the number of host immune cells using the activated strategy is $k_a$. The total number of host immune cells involved in the interaction, $K$, is:

$$K = k_b + k_a \qquad \text{Eq. S3}$$

The proportion of either $k_b$ or $k_a$ cells in the population is defined as:

$$y_j = \frac{k_j}{K} \qquad \text{Eq. S4}$$

where $j$ denotes either host strategy, $a$ or $b$.

The average payoff, $\bar{\pi}(i, y)$, or relative fitness, for a fungal cell of either type, $i$, in the presence of either host strategy, $j$, depends on the values in the payoff matrix and the proportion of the host population using a given strategy because the

proportion affects how often *C. albicans* encounters a host cell using that strategy. The average payoff is given by:

$$\bar{\pi}(i, y) = \pi(i, a)y_a + \pi(i, b)y_b \qquad \text{Eq. S5}$$

In other words, the average payoff, $\bar{\pi}$, to a *C. albicans* cell of type, *i*, depends on the chance, $y_a$, that the fungal cell contacts a host cell of type *a* and the chance, $y_b$, that it contacts a host cell of type *b*.

The population dynamics of the fungal population are given by the following two equations:

$$\dot{n_l} = n_l\bar{\pi}(l, y) + \varepsilon n_h \qquad \text{Eq. S6}$$

$$\dot{n_h} = n_h\bar{\pi}(h, y) - \varepsilon n_h \qquad \text{Eq. S7}$$

where $\dot{n_l}$ is the rate of change of the number of low Efg1p fungal cells. The first term in Equation S6 is the number of low Efg1p cells present in the population ($n_l$) multiplied by the average payoff ($\bar{\pi}$), or relative fitness, of low Efg1p cells in the presence of host cells with either host strategy, *j*, taking into account the proportion of host cells employing each strategy ($y_j$). Because *C. albicans* cells can change from high Efg1p activity to low Efg1p activity, the second term in Equation S6 is the rate of change from high to low Efg1p activity, $\varepsilon$, multiplied by the number of high Efg1p activity cells in the population ($n_h$). Including a separate term for the converse event, a change from low Efg1p activity to high Efg1p activity, did not improve the fit of the simulations to the experimental data so this event was not included in our model. Similarly, Equation S7 is the rate of change of the number of high Efg1p cells

in the fungal population. Equations S6 and S7 show that the number of *C. albicans* cells employing each type of strategy depends on the relative fitness, as given by the payoffs, and the rate of change in Efg1p activity.

The host response dynamics are governed in an analogous way:

$$\dot{k_a} = k_a \bar{\pi}(a, x) \qquad \text{Eq. S8}$$

$$\dot{k_b} = k_b \bar{\pi}(b, x) \qquad \text{Eq. S9}$$

The rate of change of host cells using the activated strategy, $a$, is equal to the number of activated host cells multiplied by their average payoff when in contact with either type of fungal cell, taking into account the proportion of the fungal population using each strategy (Equation S8). Similarly, Equation S9 defines the rate of change for naïve host cells.

The rate of change of the total size of the fungal population is defined as:

$$\dot{N} = \dot{n_l} + \dot{n_h} = N[x_l \bar{\pi}(l, y) + x_h \bar{\pi}(h, y)] \qquad \text{Eq. S10}$$

In other words, the rate of change of the entire population is equal to the sum of the rates of change of each subpopulation of fungal cells. Using the previously defined equations, Equation S10 shows that the rate of change for the whole *C. albicans* population is equal to the number of cells in the fungal population multiplied by a factor correcting for the relative fitness of each subpopulation. This factor is the proportion of low Efg1p activity cells multiplied by the average payoff for being a low Efg1p activity cell in contact with host cells using either strategy plus

the proportion of high Efg1p activity cells multiplied by the average payoff for being a high Efg1p activity cell in contact with host cells using either strategy.

Next, this equation was used to calculate the rate of change of the proportion of the fungal population with low Efg1p activity. The portion of the fungal subpopulation with low Efg1p activity is $x_l = \frac{n_l}{N}$. The rate of change of this subpopulation, $x_l$, depends on the rates of change of the number of cells in the subpopulation, $n_l$, and the number of cells in the total fungal population, $N$. From this, we formed an expression for the rate of change of the absolute number of low Efg1p activity cells:

$$\dot{n_l} = N\dot{x}_l + x_l\dot{N} \qquad \text{Eq. S11}$$

That is, the rate of change of the number of low Efg1p activity cells is equal to the number of cells in the fungal population multiplied by the change in proportion of low Efg1p cells added to the proportion of low Efg1p cells multiplied by the change in the number of cells of the fungal population. The first term represents the difference due to the change in proportion of low Efg1p activity cells in the population. The second term accounts for the change in the total population. By substituting using Equation 12 and rearranging, Equation 1 describing the rate of change of the proportion of low Efg1p activity cells was obtained:

$$\dot{x}_l = x_l(1 - x_l)[\bar{\pi}(l, y) - \bar{\pi}(h, y)] + \varepsilon(1 - x_l) \qquad \text{Eq. 1}$$

Thus, in this model, the rate of change of the proportion of low Efg1p activity cells depends on the current proportion of low Efg1p activity cells, the average

payoff for the low Efg1p strategy, the average payoff for the high Efg1p strategy, and the rate of change from high to low Efg1p activity.

A set of calculations similar to those in Equation S10 led to an expression for the rate of change of the number of host cells participating in the interaction.

$$\dot{K} = \dot{k_a} + \dot{k_b} = K[y_a\bar{\pi}(a,x) + y_b\bar{\pi}(b,x)] \qquad \text{Eq. S12}$$

In an analogous manner to Equation 1, we formed an expression for the rate of change of the proportion of the host cells active against *C. albicans*:

$$\dot{y_a} = y_a(1 - y_a)[\bar{\pi}(a,x) - \bar{\pi}(b,x)] \qquad \text{Eq. 2}$$
