# Supplementary material for: The game theory of Candida albicans colonization dynamics reveals host status-responsive gene expression
Source: BMC Syst Biol. 2016 Mar 1;10:20. doi: 10.1186/s12918-016-0268-1 (PMC4772284; doi:10.1186/s12918-016-0268-1)
Supplement: Additional file 7: Table S1. — C. albicans strains used in this study. (PDF 54 kb) [file 12918_2016_268_MOESM7_ESM.pdf]

Table S1. *C. albicans* strains used in this study

Strain	Genotype	Reference
SC5314	Wild type parent	(1)
SN100	SC5314 *Δura3::imm434/ URA3⁺* Δ*his1/Δhis1*	(2)
CKY101	SC5314 *Δura3::imm434/ Δura3::imm434 ADE2*/*ade2*::pDBI52	(3)
JPY104	SC5314 *Δura3::imm434/ Δura3::imm434 RPS1/rps1::ACT1pr-EFG1*	(4)
JPY106	SC5314 *Δura3::imm434/ Δura3::imm434 Δefg1::hisG/ Δefg1::hisG ADE2/ade2::pISAT*	(4)
JPY111	CAI-4 *Δsin3*::Sat placer/*Δsin3*::Ura placer	This study
JPY112	CAI-4 *Δsin3*/*SIN3⁺*-Ura placer	This study

References:

1. Fonzi WA & Irwin MY (1993) Isogenic strain construction and gene mapping in *Candida albicans. Genetics* (134(3):717-728.

2. Noble SM & Johnson AD (2005) Strains and strategies for large-scale gene deletion studies of the diploid human fungal pathogen *Candida albicans. Eukaryot Cell* 4(2):298-309.

3. Brown DH, Jr., Giusani AD, Chen X, & Kumamoto CA (1999) Filamentous growth of *Candida albicans* in response to physical environmental cues and its regulation by the unique *CZF1* gene. *Mol Microbiol* 34(4):651-662.

4. Pierce JV & Kumamoto CA (2012) Variation in *Candida albicans EFG1* expression enables host-dependent changes in colonizing fungal populations. *MBio* 3(4):e00117-00112.
