# Supplementary material for: The game theory of Candida albicans colonization dynamics reveals host status-responsive gene expression
Source: BMC Syst Biol. 2016 Mar 1;10:20. doi: 10.1186/s12918-016-0268-1 (PMC4772284; doi:10.1186/s12918-016-0268-1)
Supplement: Additional file 8: Table S2. — Primers used in this study. (PDF 50 kb) [file 12918_2016_268_MOESM8_ESM.pdf]

Table S2. Primers used in this study.

A. qRT-PCR primers

Gene	Primer Name	Primer Sequence
ACT1	ACT1 163F	GTT GGT GAT GAA GCC CAA TC
ACT1	ACT1 241R	CCC AGT TGG AAA CAA TAC CG
EFG1	EFG1 1155F	AAA TAC CAA GGC TGC CAC TG
EFG1	EFG1 1195R	TAC CTT GAG GGA TAC CAG CAG
YNG2	YNG2 456F	GAA TGG ATC ATC GGC AAC TC
YNG2	YNG2 571R	CCT GTC TTG AAG CAG AGG ATG
ESA1	ESA1 1340F	GGC TGG GAC TTA ATT CAT CG
ESA1	ESA1 1419R	CGT GGT CAT GCA TGT TAT GG
SAS2	SAS2 670F	CCC CCG TTT CAA AGA AGA C
SAS2	SAS2 764R	GGG CCA CTG CTT TTA AGT TG
PMA1	PMA1 2567f	CCG AAG CCT TTG ACA ACT TC
PMA1	PMA1 2659r	CTC TTT GCA TGG ACA CAA GG
LSC2	LSC2 804f	AGC TGA AGC CGG TAA ATA CG
LSC2	LSC2 884r	GCC AAA CCA GCA CCA TTA AC
HDA1	HDA1 2137F	GCG TTT CTA AAC CGA ACG AC
HDA1	HDA1 2280R	ATT GCC ATT ACC AGC ACC AC
TUP1	TUP1 1151F	ATG GCC ACG AAG ATT CAG TC
TUP1	TUP1 1250R	TCC AAG TGC CAC AAC TTG AC
SET3	SET3 2891F	CAA CAG TCC AAC AAC CAA CC
SET3	SET3 2964R	TGG GAC AGG AAT CTC AAC TG
HOS2	HOS2 1214F	ATG CTC CAA GTG TGC AAA TG
HOS2	HOS2 1319R	TCA TCT CGC TCC ATG TCT TC
SIN3	SIN3 4066F	CCA GAC GCA AAA TCA GCA AC
SIN3	SIN3 4214R	TGG GCT TTG TCA TTC TCT CC
RPD3	RPD3 905F	CCG TGA TGG TAT TAG GTG GTG
RPD3	RPD3 981R	GCA AAC TCC GGT TTC AAA TG

FLO8	FLO8 2006F	CAA CTC AAG CCA CTG CTA TGT C
FLO8	FLO8 2123R	TGC CCA CGA TTA TGA TTG C
TPK1	TPK1 1003F	CAA GGT GGT TCT GAT GAT GTG
TPK1	TPK1 1130R	TGT GAA GTA TCA CCA ACA CCT G
RAS1	RAS1 709F	GGA GGT GTT TCT TCT GGT CAA G
RAS1	RAS1 797R	TGT GGT TCT TGT TGT TGC TG
BCY1	BCY1 914F	GTT GGG CAT TAG ACC GTT TG
BCY1	BCY1 1037R	CGT GCA TGA TCC GAA AGA G
PDE2	PDE2 1361F	CTG CCT TGT TAA TCA AAT GTG C
PDE2	PDE2 1431R	TGC CCA TTG TGC AGA TAC TC
IL-17A	Forward	GCTCCAGAAGGCCCTCAGA
IL-17A	Reverse	AGCTTTCCCTCCGCATTGA
IL-22	Forward	ATCGTCAACCGCACCTTTAT
IL-22	Reverse	GACTCCTCGGAACAGTTTCTC
GAPDH	Forward	TGCATGGCCGTTCTTAGTTG
GAPDH	Reverse	AGTTAGCATGCCAGAGTCTCGTT

B. SIN3 primers

SIN3A	TGTAGGGTTTTAGAATGCAGTATGGTAGTT
SIN3B	agctttGTTTAAACTTTTTGAACGGGGGAGGAGATT
SIN3C	ttgGCGCGCATGACAACAACACAGAACCAGAC
SIN3D	AACTACCATACTGCATTCTAAAACCCTACA
	agatctTGTCAAAGATTAAATCAAAGAGCA
Sn3S3F	CAATTAAAAATGGGAAAAGCATGAATAGAACTG
Sn3S3R	GTCGCACACGCACCGAGGCAAAG
Sn3U5F	GCATTTTGCAAAGCTTCTCGTGGTG
Sn3U5R	TGCCGTGCAAGTTTCTATCTCCTCCTCCTCCTAT
Sn3OF	ACACAACTATTTATGTTTTCTTCCGTCACTT
Sn3OR	TTTGTAGGTTCATTAGCCTCCGATTCT

Sin3cF	ttggcgcgcGTAGCCCATATTCATTATAAAACAAGAG
Sin3cR	agctttgtttaaacTTAAAAATGGGAAAAGCATGAATAGAACT
